# DSN1 drives breast cancer progression via cell cycle regulation: diagnostic and therapeutic implications

**DOI:** 10.3389/fonc.2025.1711214

**Published:** 2026-01-20

**Authors:** Dongyang Liu, Manuel A. Luis, Djojomoenawi Sherilyn H. G., Xiaoxuan Zhu, Xiuqin Zhang, Yuan Fang, Xiaoyan Lin, Shasha Tang, Fengfeng Cai

**Affiliations:** 1 Department of Breast Surgery, Tongji Hospital, School of Medicine, Tongji University, Shanghai, China; 2 Department of Pathology, Tongji Hospital, School of Medicine, Tongji University, Shanghai, China; 3 Department of Breast Surgery, Yangpu Hospital, School of Medicine, Tongji University, Shanghai, China

**Keywords:** breast cancer, cell cycle, cell proliferation, drug sensitivity, DSN1

## Abstract

**Aim:**

Breast cancer is the most prevalent form of cancer among females and carries a substantial societal impact. DSN1, a component of the MIS12 complex, plays a critical role in centromere assembly, distribution, and stability. While DSN1’s role in tumors has been investigated, its specific function in breast cancer remains unclear.

**Methods:**

First, we utilized bioinformatics techniques to explore DSN1 expression in breast cancer and conducted functional enrichment and correlation analyses. Subsequently, we assessed the clinical relevance of DSN1 through immunohistochemistry. Furthermore, we examined how DSN1 affects the growth of breast cancer cells by conducting CCK8 and colony formation tests. Cell cycle and apoptosis changes were assessed using flow cytometry. Moreover, we examined key genes related to cell cycle and apoptosis to further elucidate the underlying mechanisms. Finally, we screened potential drugs targeting DSN1 by drug sensitivity and molecular docking analyses.

**Results:**

Bioinformatics analysis revealed that DSN1 is highly expressed in breast cancer, making it a potential diagnostic marker. Functional enrichment analysis indicated that the DSN1- overexpressed group was enriched in cell proliferation-related pathways. Cellular experiments confirmed that DSN1 promotes breast cancer proliferation by affecting cell cycle pathways, involving key molecules such as CCNB1, CCND1, CKD1, CDK4, and CDK6. Drug sensitivity analysis showed that the DSN1 high expression group was resistant to drugs such as Epirubicin, Cyclophosphamide, Ribociclib, and Palbociclib, but relatively sensitive to tamoxifen and lapatinib.

**Conclusions:**

DSN1 contributes to breast cancer progression by modulating cell cycle pathways, making it a potential diagnostic and therapeutic target with clinical applicability.

## 1 Introduction

Breast cancer is the most prevalent cancer among women, causing the highest number of cancer-related deaths in this demographic and presenting a significant societal challenge (1, 2). Despite advancements in molecular subtyping and classification for treatment, many patients still face poor prognoses (3, 4). Therefore, it is crucial to deepen our understanding of breast cancer’s causes and progression, identify potential therapeutic targets, and elucidate their mechanisms to improve detection and management.

DSN1, also known as the DSN1 component of the MIS12 kinetochore complex, encodes a kinetochore protein that is part of the minichromosome instability-12 kinetochore complex (5–7). It is essential for normal chromosome alignment, segregation, and kinetochore formation during mitosis (5). This protein is vital for proper kinetochore assembly and cell cycle progression (8). Alternative splicing of DSN1 results in multiple transcript variants. Recent studies link increased DSN1 levels to chromosomal instability (CIN), a key factor in cancer development, with 80% of tumors exhibiting CIN (9, 10). Nevertheless, there has been a lack of extensive studies on the role and biological impact of DSN1 in cancer. Studies have revealed overexpression of DSN1 in colorectal cancer, influencing CRC cell migration and proliferation (11). Another study has shown that DSN1 is strongly linked to clinical pathological characteristics and can function as a standalone prognostic indicator for liver cancer (12, 13). Recent advances have uncovered DSN1’s multifaceted roles in cancer. Research shows that Fucoxanthin displays strong anti-tumor activity in models of colorectal cancer by suppressing DSN1 expression, hinting that DSN1 could be a new drug target (14). Other research demonstrates that DSN1 levels are significantly higher in low-grade gliomas (LGGs) than in normal brain tissue. Low expression and hypermethylation (a high level of methylation) of DSN1 are linked to much longer patient survival, making DSN1 a risk factor with high diagnostic and prognostic value for LGG patients (15).Nevertheless, its role and significance in breast cancer remain unclear.

To explore DSN1’s potential significance in breast cancer, we first analyzed its expression and clinical relevance using the Cancer Genome Atlas (TCGA) database. We then conducted GO and GSEA enrichment analyses to investigate the functions and pathways highly correlated with DSN1. Additionally, to better understand DSN1’s potential impact, we assessed its clinical significance through immunohistochemistry and conducted *in vitro* studies to elucidate its role and mechanisms in breast cancer. Furthermore, we screened potential drugs targeting DSN1 by drug sensitivity and molecular docking analyses.

Our findings indicate that DSN1 is overexpressed in breast cancer and that higher levels of DSN1 are associated with adverse clinical pathological features. Enrichment analysis suggests that DSN1 is involved in cell division pathways. *In vitro* studies confirm that DSN1 is abundantly present in breast cancer and influences cell growth by affecting the cell cycle pathway. Drug sensitivity and molecular docking analyses suggest that Nutlin-3a, Lapatinib and Tamoxifen are potentially effective agents for DSN1-overexpressing breast cancers. These results suggest that DSN1 could serve as a valuable marker for breast cancer detection and management.

## 2 Methods

### 2.1 Bioinformatics analysis of DSN1 expression and function

Breast cancer (BC) expression data and associated clinical information were obtained from The Cancer Genome Atlas (TCGA-BRCA) project. The analysis included 1098 BC patients, with clinical data for patients with unknown profiles treated as missing values. Since all data used in this study were sourced from the TCGA database, ethical approval and informed consent were not required.

Differential gene expression analysis between high and low DSN1 expression groups in BC was performed using the DESeq2 package on HTSeq-counts expression profiles (16). The criteria for identifying differentially expressed genes (DEGs) were |log2FoldChange| > 2 and p.adj < 0.05. Spearman’s correlation analysis was employed to establish relationships between DSN1 and other genes.

Metascape (https://metascape.org/) is a valuable platform for gene list annotation and analysis, particularly for experimental biologists (17). In this study, Metascape was used to conduct enrichment analysis of DSN1-associated DEGs in BC, with thresholds set at p < 0.01, a minimum count of 3, and an enrichment factor > 1.5.

Gene Set Enrichment Analysis (GSEA) was carried out using the clusterProfiler package in R version 3.6.2 to investigate the roles and pathways of DSN1-associated DEGs in BC (18). Groups were categorized based on DSN1 expression levels, with both low and high levels identified. Each analysis included 1000 permutations of gene sets. DSN1 expression level was used as the phenotype label, and results were arranged according to an adj. p-value < 0.05 and FDR q-value < 0.25 for each phenotype.

Statistical analyses were conducted using R software version 3.6.2. Wilcoxon signed-rank tests were employed to evaluate DSN1 expression in unpaired samples, and the pROC package was used to calculate the area under the ROC curve to assess the discriminatory ability of DSN1 in BC (19). Pearson’s chi-square and Fisher’s exact tests were used to analyze the correlation between DSN1 expression and clinicopathologic factors.

### 2.2 Cell culture, reagents, and tissue samples

The human breast cancer cell lines MCF-7, MDA-MB-231, MDA-MB-453, MDA-MB-468, BT-549, and SKBR-3, along with the normal human breast epithelial cell line MCF10A, were obtained from the Chinese Academy of Science in Shanghai, China. The breast cancer cell lines were cultured in DMEM high glucose medium (Gibco, Thermo Fisher Scientific, Waltham, MA, USA), whereas MCF10A cells were maintained in RPMI-1640 medium (Gibco, USA) and mammary epithelial cell medium (Procell, Wuhan, China). All growth media were supplemented with 10% fetal bovine serum (Gibco, USA), 100 U/mL penicillin, and 100 μg/mL streptomycin (Servicebio, Wuhan, China). Cell passaging was performed using a 0.25% trypsin-EDTA solution (Servicebio, China). The cell lines were kept in an incubator at 37°C with 5% CO2.

Breast cancer tissue samples and normal tissues were collected from the Breast Surgery Department at Shanghai Tongji Hospital immediately after surgery and stored at -80°C. Written consent was obtained from all participants, with approval from the Medical Ethics Committee of Shanghai Tongji Hospital (No.2024-DW-SB-025). Histopathological confirmation was performed on all tissue samples, and their clinical data were collected accordingly.

### 2.3 Immunohistochemical staining

Paraffin-embedded tissues were sectioned, deparaffinized, rehydrated, and subjected to antigen retrieval using sodium citrate. The sections were then blocked and incubated with a DSN1 antibody (1:200; Santa Cruz Biotechnology), followed by an HRP-labeled anti-rabbit secondary antibody, and stained with diaminobenzidine (DAB) for immunohistochemistry. Staining intensity was scored as 0 (negative), 1 (weak), 2 (moderate), or 3 (strong). The proportion of positive tumor cells was scored as 0 (0%), 1 (1–25%), 2 (26–50%), 3 (51–75%), or 4 (76–100%). The final IHC score was calculated by multiplying the intensity and proportion scores. Two independent pathologists evaluated DSN1 expression based on cell proportions and staining intensity, categorizing patients into low and high expression groups according to IHC scores, using the median value as the cutoff.

### 2.4 Analysis using quantitative real-time PCR

Breast cancer tissues and cells were used to extract total RNA with TRIzol^®^ Reagent (Invitrogen, USA), then converted into cDNA for qPCR using HiScript^®^ III RT SuperMix (Vazyme, Shanghai, China). Subsequently, quantitative real-time PCR was conducted on the LightCycler^®^ 96 Instrument (Roche Diagnostics) with 2 × Taq Pro Universal SYBR qPCR Master Mix (Vazyme, Shanghai, China). The specific primers used for the experiment were as follows: DSN1 forward, 5′- CCGGTCTATCAGTGTCGATTTAG -3′ and DSN1 reverse, 5′- TGTCCCTTAGGAAAGGTTCAAG -3′; CCNB1 forward, 5′- CAGTTCCGACTCATTATGTCTTCC -3′ and CCNB1 reverse, 5′- TCCCTTCTTCTTGTTGCTTCCA -3′; CCND1 forward, 5′- GTCCTACTTCAAATGTGTGCAG -3′ and CCND1 reverse, 5′- GGGATGGTCTCCTTCATCTTAG -3′; CDK1 forward, 5′- GGTTCCTAGTACTGCAATTCG -3′ and CDK1 reverse, 5′- TTTGCCAGAAATTCGTTTGG -3′; CDK4 forward, 5′- ATGTGGAGTGTTGGCTGTATC -3′ and CDK4 reverse, 5′- CAGCCCAATCAGGTCAAAGA -3′; CDK6 forward, 5′- GACCAGCAGCGGACAAATA -3′ and CDK6 reverse, 5′- TGACGACCACTGAGGTTAGA -3′; BAX forward, 5′- TGAAGACAGGGGCCTTTTTG -3′ and BAX reverse, 5′- AATTCGCCGGAGACACTCG -3′; BCL2 forward, 5′- GTGGATGACTGAGTACCTGAAC -3′ and BCL2 reverse, 5′- GAGACAGCCAGGAGAAATCAA -3′; CASP3 forward, 5′- TGGTGATGAAGGGGTCATTTATG -3′ and CASP3 reverse, 5′- TTCGGCTTTCCAGTCAGACTC -3′; CASP9 forward, 5′- CTGTCTACGGCACAGATGGAT -3′ and CASP9 reverse, 5′- GGGACTCGTCTTCAGGGGAA. GADPH forward, 5′- CATTGACCTCAA CTACATGGTTT -3′ and GADPH reverse, 5′- GAAGATGGTGATGGGATTTCC -3′; GADPH was used as an internal control and results are shown as relative expressions calculated by the 2−ΔΔCT method.

### 2.5 Cell transfection

Two custom-designed human DSN1 siRNA sequences (RNAi1: 5′-GGATGAACTGCAAGGAT-3′ and RNAi2: 5′-GGGATCAGCTCTTGCTTCA-3′) were developed to knock down DSN1 expression. A non-targeting siRNA served as the negative control. Additionally, the plasmids pENTER and pENTER-CMV-DSN1 (DSN1 plasmid) were used to overexpress DSN1. All sequences were synthesized by Generay Biotechnology (Shanghai, China) and transfected into cells in 6- or 96-well plates using Lipofectamine^®^ 3000 reagent (Invitrogen, Thermo Fisher Scientific, Waltham, MA, USA) following the manufacturer’s protocol.

### 2.6 Cell proliferation detection for cell viability assay and colony formation assay

Cell viability post-transfection with siRNAs and plasmids was assessed using the Cell Counting Kit 8 (CCK-8; Dojindo Molecular Technologies, Inc., Kumamoto, Japan). Cells were plated at a density of 8000 cells per well in a 96-well plate and incubated at 37°C for 48 hours post-transfection. Each treatment was performed in triplicate. Subsequently, 10 µL of CCK-8 solution was added to each well, and the cells were incubated for an additional 2 hours. The optical density at 450 nm was measured using a SpectraMax i3 instrument (Molecular Devices, San Jose, CA, USA). This experiment was repeated three times.

Furthermore, cells successfully transfected for 48 hours were transferred to 6-well plates at a concentration of 200 cells per well. After 7 days of culture, the cells were washed three times with phosphate-buffered saline, fixed with 4% paraformaldehyde for 10 minutes, and stained with 0.5% crystal violet for 5 minutes. The wells were then rinsed with water and examined microscopically.

### 2.7 Flow cytometry analysis for cell cycle distribution and apoptosis

Following trypsin digestion, the cells were harvested and resuspended in chilled PBS, then fixed in 75% ethanol overnight. After three washes with PBS and centrifugation, the cells were treated with a solution containing 50 g/ml propidium iodide (PI) and RNA enzyme at 37°C for 1 hour. Flow cytometry was used to analyze the cell cycle distribution across different groups. At 72 hours post-transfection, apoptosis was measured using an Annexin V-FITC/PI Apoptosis Kit. Cells were washed with PBS and resuspended in a binding buffer containing Annexin V-FITC and PI. After a 15-minute incubation, flow cytometry was performed to determine the apoptosis rate.

### 2.8 Drug sensitivity and molecular docking analyses of DSN1

To explore therapeutic drugs targeting DSN1, we first searched the drug sensitivity of DSN1 in pan-cancer in GSDC data by the online analysis platform GSCA (https://guolab.wchscu.cn/GSCA). Then, the drug sensitivity prediction was performed using the gene expression profile of the TCGA-BC cohort as the input dataset. The oncoPredict algorithm then estimated the IC50 values by comparing the tumor transcriptomes to pre-trained models derived from the GDSC database (20). Molecular docking was performed using AutoDock Vina v1.2.3 to predict interactions between DSN1 and potential therapeutic agents. The DSN1 protein structure was prepared by removing water molecules and adding polar hydrogens. Ligand structures were optimized with Avogadro using the MMFF94 force field. A docking grid (25 × 25 × 25 Å) centered on the DSN1 active site (coordinates: X = 10.5, Y = 15.2, Z = 20.1) was defined to cover all potential binding pockets. For each ligand, the top 10 binding poses were generated and ranked by binding energy (kcal/mol), with a threshold of ≤−6.0 kcal/mol considered significant (21). The lowest-energy conformation was selected for visualization in PyMOL, and pose stability was validated by root-mean-square deviation (RMSD) analysis (<2.0 Å across three independent runs) (22).

### 2.9 Statistical analysis

The experiment was repeated three times independently, and results are expressed as the mean ± standard deviation from these tests. Statistical analysis was conducted using t-tests or one-way ANOVA with GraphPad Prism version 9.0, considering a p-value less than 0.05 as statistically significant.

## 3 Results

### 3.1 The expression of DSN1 and its correlation with clinical parameters.

**Figure 1:**
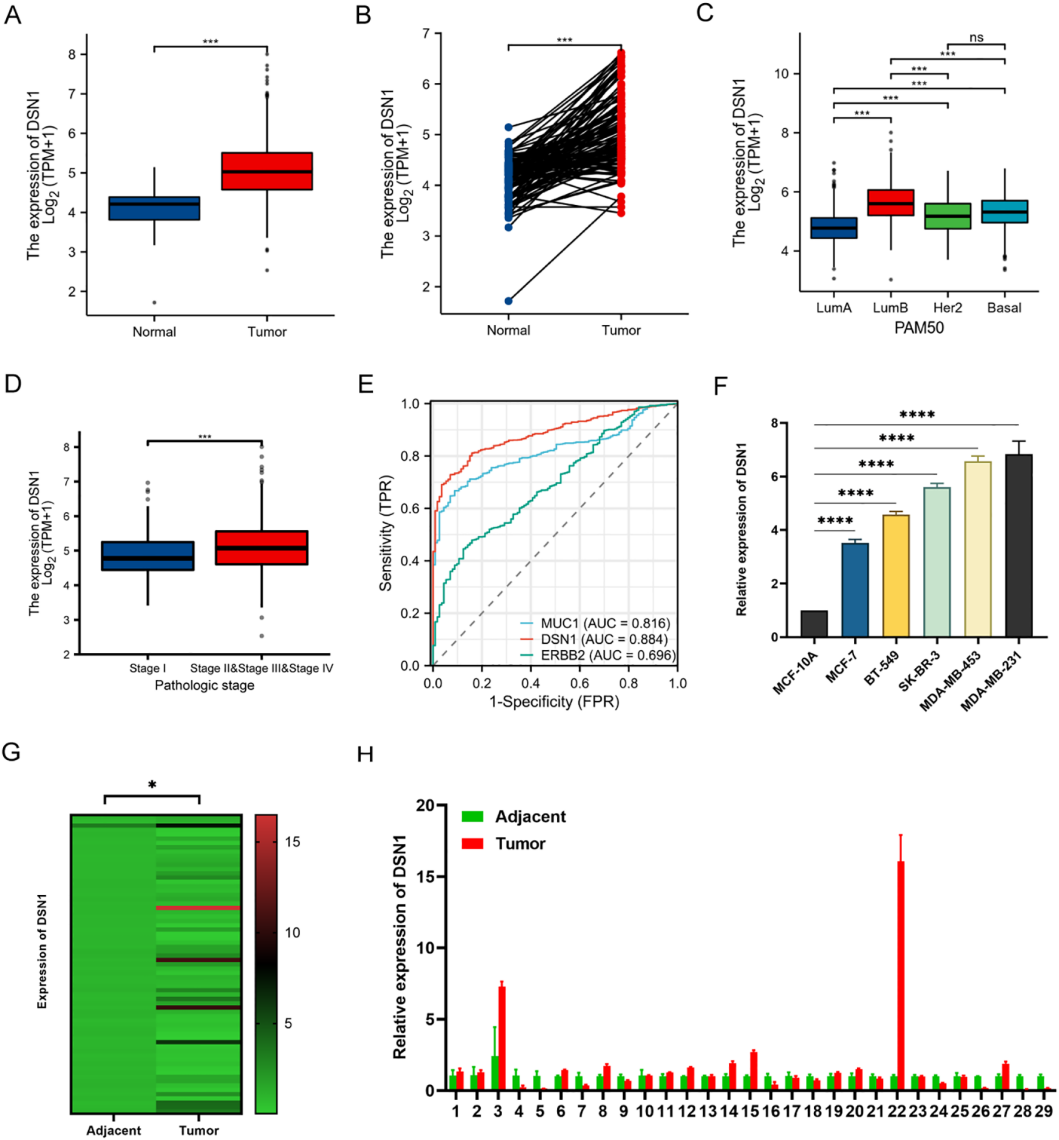
DSN1 expression in breast cancer (BC) and their relevance with clinical characteristics. **(A)** Relative expression of DSN1 mRNA in normal (n = 113) and tumor tissues (n = 1113) in BC. **(B)** Relative expression of DSN1 mRNA in paired tumor and normal samples (n = 113) in BC. **(C)** Relative expression of DSN1 mRNA across molecular subtypes of BC. Note that a subset of tumor samples shows lower DSN1 mRNA expression compared to matched normal tissues, possibly reflecting tumor heterogeneity. **(D)** Relative expression of DSN1 mRNA in Stage I vs. Stages II–IV tumors in BC. **(E)** ROC curves were generated for the gene DSN1, MUC1 and ERBB2 to differentiate between BC samples and normal samples. The abscissa represents False Positive Rate, the ordinate represents Ture Positive Rate. **(F)** Relative DSN1 mRNA expression in normal mammary epithelial cells (MCF-10A) and breast cancer cells. **(G)** Heatmap of DSN1 mRNA expression across 69 breast cancer tissues and normal controls. **(H)** Relative DSN1 mRNA levels in paired tumor and normal tissues from 29 breast cancer patients. Significance levels are denoted as follows: *p < 0.05, ***p < 0.001, ****p < 0.0001.

DSN1 expression was significantly upregulated in breast cancer (p < 0.001) and was markedly higher in tumor tissues compared to normal tissues (p < 0.001) (**Figures 1A, B**). Expression levels varied among breast cancer subtypes, with the highest observed in luminal B (**Figure 1C**). Moreover, DSN1 levels were elevated in advanced-stage patients (**Figure 1D**). ROC curve analysis demonstrated the diagnostic potential of DSN1 in BC, with an AUC of 0.885 (95% CI: 0.862–0.907), over CA153 (MUC1, AUC = 0.816) and HER2 (ERBB2, AUC = 0.696) suggesting that DSN1 could serve as a biomarker for distinguishing cancer patients from controls (**Figure 1E**). Furthermore, survival analysis using the TCGA cohort revealed that high DSN1 expression significantly predicted inferior overall survival (P = 0.049; **Supplementary Figure S2A**). This association was particularly evident in luminal A and B subtypes (P = 0.011, **Supplementary Figure S2B**), underscoring its prognostic value in hormone receptor-positive breast cancer.

To verify DSN1 expression levels in breast cancer cells and tissues, quantitative real-time polymerase chain reaction (qRT-PCR) analyses were performed. Results showed increased DSN1 expression in breast cancer cell lines (MCF-7, BT-549, SK-BR-3, MDA-MB-453, and MDA-MB-231) compared to normal breast epithelial cells (MCF10A) (**Figure 1F**). Furthermore, DSN1 expression was assessed in 69 breast cancer tissues and normal tissues, revealing significant upregulation in cancer tissues (**Figure 1G**). Similarly, analysis of 29 paired tissues confirmed elevated DSN1 levels in breast cancer tissues, although a subset of tumors exhibited lower expression than their normal counterparts, which may indicate molecular heterogeneity within breast cancers. While individual variations exist, the collective analysis confirms elevated DSN1 in breast cancer tissues (p=0.002) (**Figure 1H**). These findings collectively indicate a strong presence of DSN1 in breast cancer cells and tissues, suggesting a potential role in disease progression.

### 3.2 High levels of DSN1 are linked to adverse clinicopathological features in breast cancer

**Figure 2:**
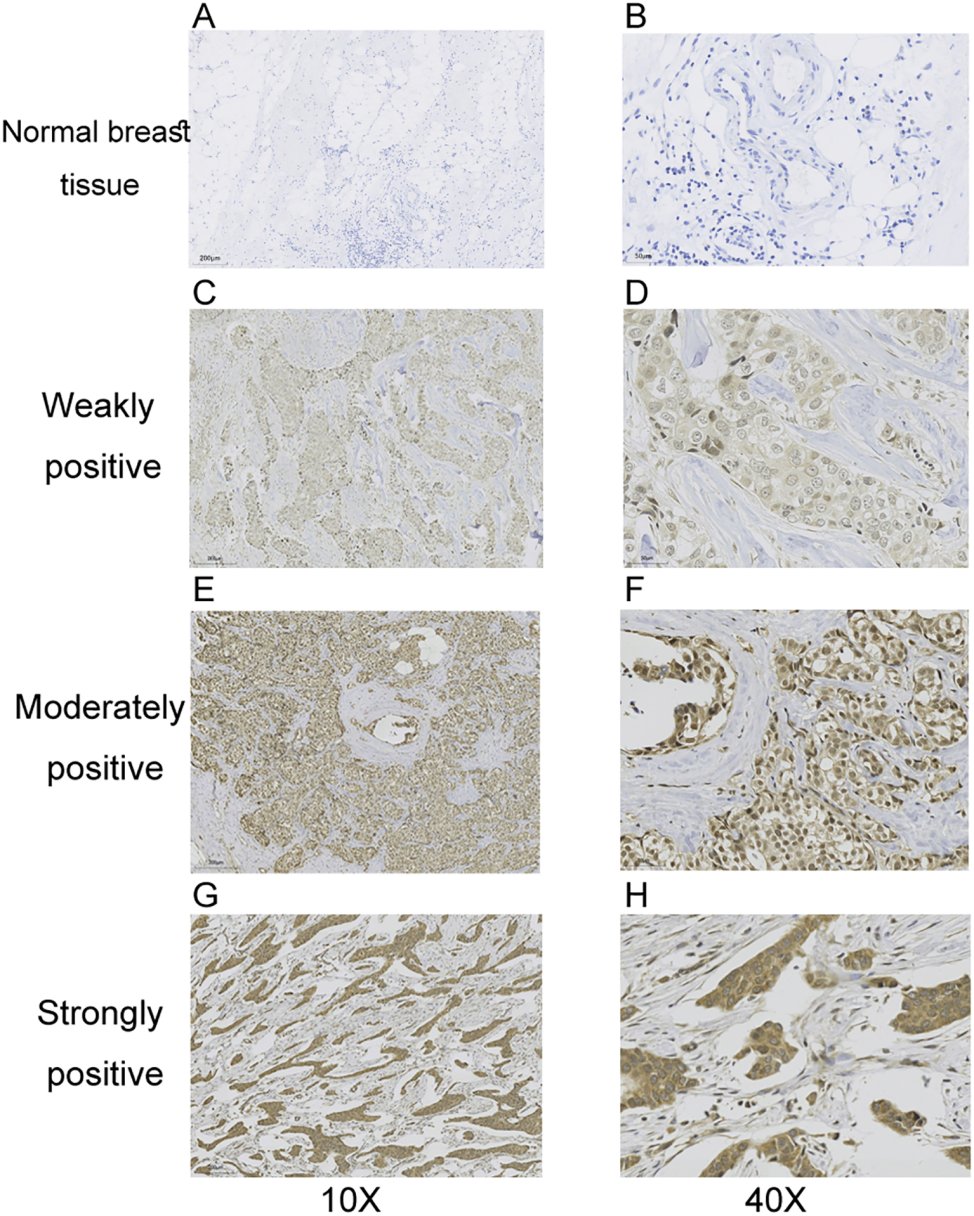
Representative immunohistochemical images of DSN1 expressed in breast cancer tissues. **(A, B)** DSN1 expressed images in normal breast tissues, at 10X and 40X magnification respectively. **(C, D)** DSN1 weakly expressed images in breast cancer tissues, at 10X and 40X magnification respectively. **(E, F)** DSN1 moderately expressed images in breast cancer tissues, at 10X and 40X magnification respectively. **(G, H)** DSN1 strongly expressed images in breast cancer tissues, at 10X and 40X magnification respectively. Statistical correlations between DSN1 expression and clinical features are provided in **Table 1**.

To investigate the relationship between DSN1 expression and clinical significance in breast cancer, immunohistochemistry was utilized to assess DSN1 levels in 61 breast cancer tissue samples. The various expression profiles of DSN1 are depicted in **Figure 2**. After reviewing the patient’s clinical and pathological information, clinical correlations are summarized in **Table 1**.We found a notable correlation between DSN1 expression and N classification (p = 0.024), molecular classification (p = 0.021), and ki67 classification (p < 0.001). Notably, no correlation was observed between DSN1 expression and the remaining clinical parameters.

**Table 1:** Correlation between DSN1 expression and clinicopathologic characteristics of breast cancer patients.

Characteristics	Total (n = 61)	DSN1		*p* value
		Low (n = 21)	High (n = 40)	
Age, n (%)				0.105
<65 y	38 (62.3)	16 (76.2)	22 (55)	
>=65 y	23 (37.7)	5 (23.8)	18 (45)	
Menopausal, n (%)				0.751
No	13 (21.3)	5 (23.8)	8 (20)	
Yes	48 (78.7)	16 (76.2)	32 (80)	
Tumor location, n (%)				0.355
Left	27 (44.3)	11 (52.4)	16 (40)	
Right	34 (55.7)	10 (47.6)	24 (60)	
Grade, n (%)				0.217
I	5 (8.2)	1 (4.8)	4 (10)	
II	41 (67.2)	12 (57.1)	29 (72.5)	
III / IV	15 (24.6)	8 (38.1)	7 (17.5)	
T classification, n (%)				0.259
T1	24 (39.3)	11 (52.4)	13 (32.5)	
T2	36 (59.0)	10 (47.6)	26 (65)	
T3	1 (1.6)	0 (0)	1 (2.5)	
N classification, n (%)				**0.024**
N0	51 (83.6)	14 (66.7)	37 (92.5)	
N1-N3	10 (16.4)	7 (33.3)	3 (7.5)	
ER status, n (%)				0.098
Negative	18 (29.5)	9 (42.9)	9 (22.5)	
Positive	43 (70.5)	12 (57.1)	31 (77.5)	
PR status, n (%)				0.098
Negative	18 (29.5)	9 (42.9)	9 (22.5)	
Positive	43 (70.5)	12 (57.1)	31 (77.5)	
HER2 status, n (%)				0.907
Negative	43 (70.5)	15 (71.4)	28 (70)	
Positive	18 (29.5)	6 (28.6)	12 (30)	
Molecular classification, n (%)				**0.021**
luminal A	25 (41.0)	4 (19)	21 (52.5)	
luminal B	9 (14.8)	5 (23.8)	4 (10)	
HER2-enriched	18 (29.5)	6 (28.6)	12 (30)	
Triple negative	9 (14.8)	6 (28.6)	3 (7.5)	
ki67 classification, n (%)				**< 0.001**
<= 14%	23 (37.7)	16 (76.2)	7 (17.5)	
> 14%	38 (62.3)	5 (23.8)	33 (82.5)	

Bold values indicate statistically significant differences (p < 0.05).

### 3.3 Functional enrichment analysis and GSEA identify significant terms and pathways associated with DSN1 in BC

**Figure 3:**
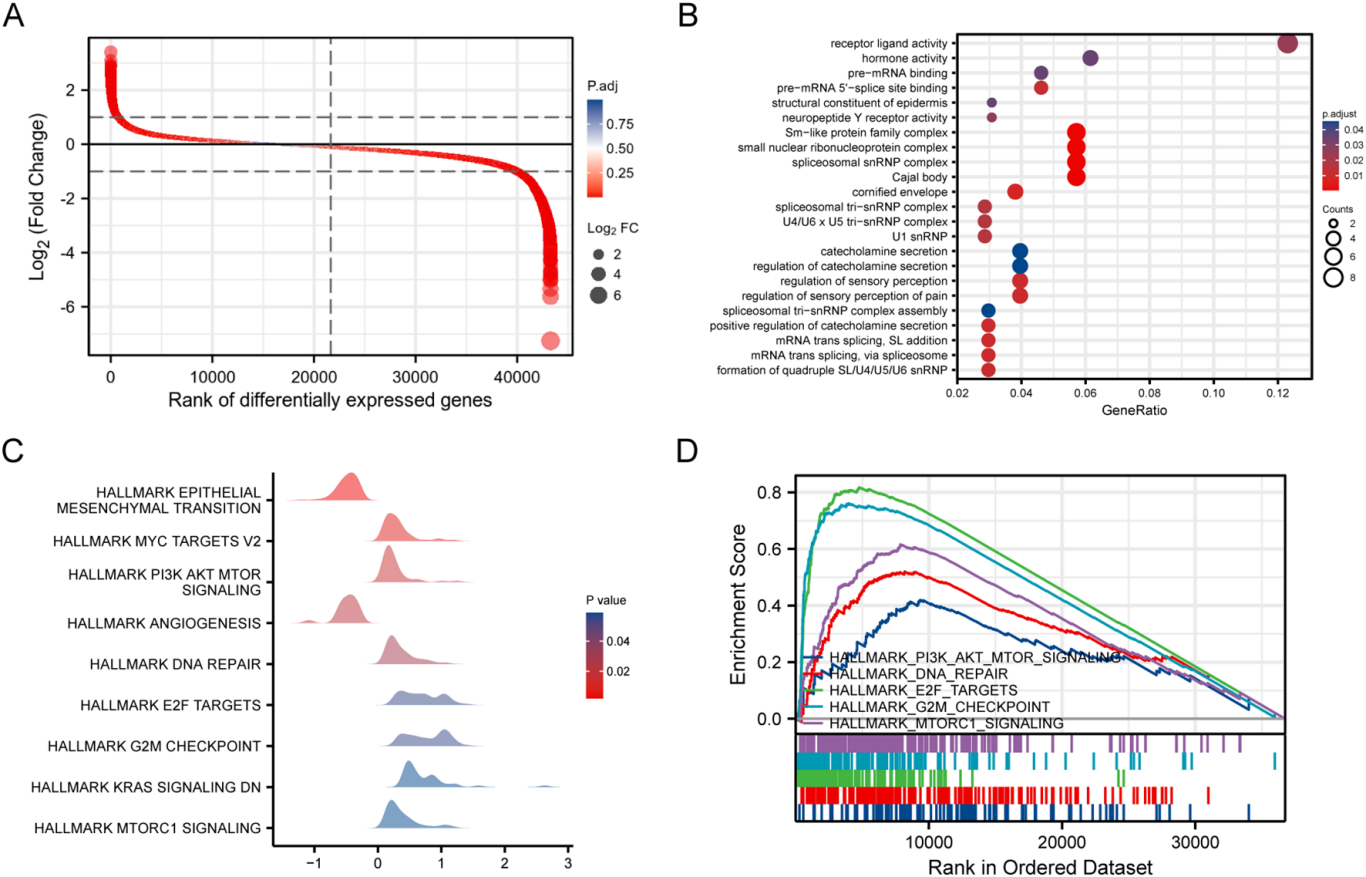
Identification of genes with differential expression between groups with high and low DSN1 expression, functional enrichment analysis of these genes, and gene set enrichment analysis (GSEA) of genes related to DSN1. **(A)** Differential ranking plot of differentially expressed genes (DEGs) between high and low DSN1 expression groups, include 110 upregulated and 983 downregulated. **(B)** Heatmaps of the GO annotations enriched in DEGs. The bar color shade was decided by the P value, the darker indicates a smaller p-value. **(C)** Mountain chart displaying the results of gene set enrichment analysis (GSEA). GSEA plot for one representative Hallmark gene set significantly enriched in the DSN1-high group. **(D)** GSEA-enrichment plots of representative Hallmarkers.

Differential expression analysis between high and low DSN1 expression groups, performed using the DESeq2 R package, identified a total of 1,093 DSN1-associated differentially expressed genes, with 110 upregulated and 983 downregulated (**Figure 3A**). Metascape analysis of Gene Ontology (GO) annotations and pathways for these genes in BC indicated involvement in processes such as receptor ligand activity, mRNA trans splicing via spliceosome, and pre-mRNA 5’-splice site binding (**Figure 3B**).

To further elucidate notable terms and pathways impacted by DSN1 in BC, Gene Set Enrichment Analysis (GSEA) against the Molecular Signatures Database (MSigDB) Hallmark gene sets was performed to identify biological pathways associated with DSN1 expression. The results, partially shown in **Figures 3C, D**, revealed that the high DSN1 expression group exhibited enrichment in MYC targets V2, PI3K-AKT-MTOR signaling, E2F targets, and G2M checkpoint pathways (**Figure 3C**). A summary of the key GSEA findings is presented in **Figure 3D**, suggesting a potential role of DSN1 in influencing the cell cycle and promoting cell proliferation in BC.

### 3.4 Identification of DSN1 correlation genes in BC

**Figure 4:**
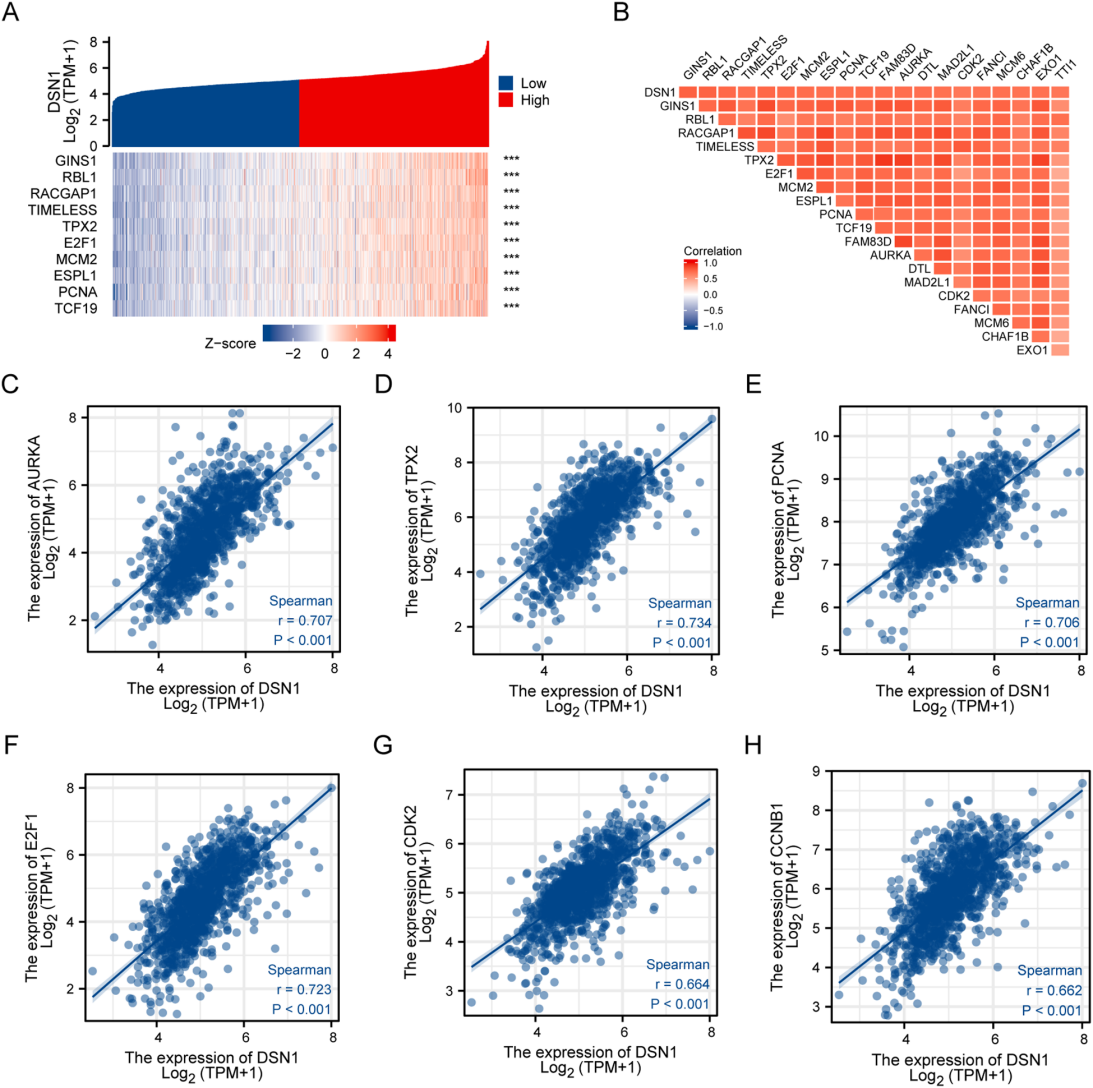
DSN1 correlation genes in breast cancer (BC). **(A)** Expression levels of the top 10 genes positively correlated with DSN1 in high and low DSN1 expression groups. Heatmap depicting normalized gene expression values (Z-score) of the top 10 positively correlated genes across samples stratified by DSN1 expression. **(B)** Matrix of correlation coefficients between DSN1 and the top 20 positively correlated genes. Heatmap of pairwise Spearman correlation coefficients (r) for the top 20 positively correlated genes. **(C)** Correlation of the expression of DSN1 and the expression of AURKA in BC. **(D)** Correlation of the expression of DSN1 and the expression of TPX2 in BC. **(E)** Correlation of the expression of DSN1 and the expression of PCNA in BC. **(F)** Correlation of the expression of DSN1 and the expression of E2F1 in BC. **(G)** Correlation of the expression of DSN1 and the expression of CDK2 in BC. **(H)** Correlation of the expression of DSN1 and the expression of CCNB1 in BC.

A study was conducted to investigate the correlation between DSN1 expression and related genes in BC. The top 10 genes correlated with DSN1 are visualized in **Figure 4A**, while a heatmap displaying the top 20 correlated genes with DSN1 is shown in **Figure 4B** DSN1 exhibited a strong positive relationship with AURKA (r = 0.707, p < 0.001, **Figure 4C**), TPX2 (r = 0.734, p < 0.001, **Figure 4D**), PCNA (r = 0.706, p < 0.001, **Figure 4E**), E2F1 (r = 0.723, p < 0.001, **Figure 4F**), CDK2 (r = 0.664, p < 0.001, **Figure 4G**), and CCNB1 (r = 0.662, p < 0.001, **Figure 4H**). These genes are all associated with cell proliferation.

### 3.5 DSN1 promotes breast cancer cell proliferation

**Figure 5:**
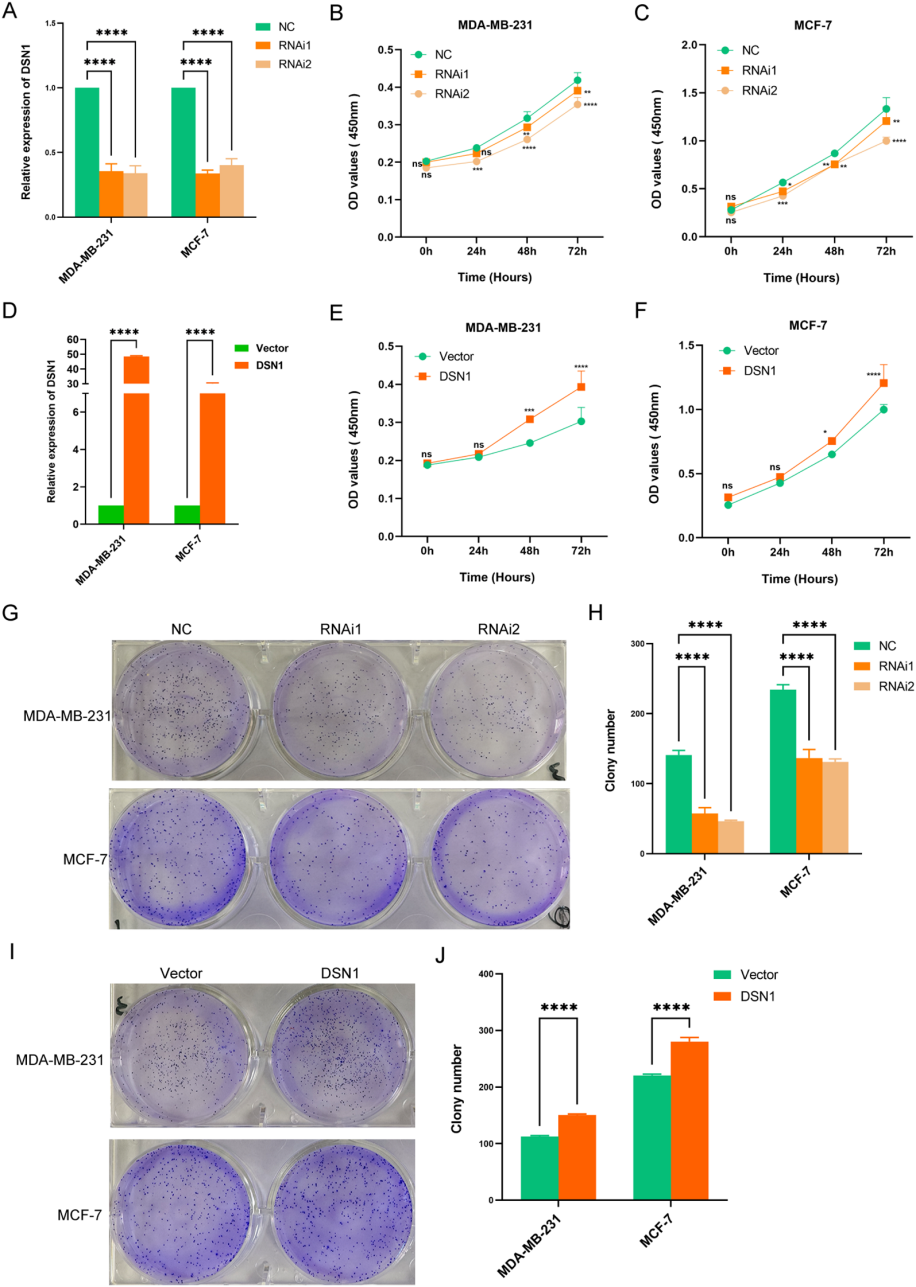
DSN1 modulates proliferation of breast cancer cells. **(A–C)** CCK-8 cell viability assay after DSN1 downregulation on breast cancer cell lines MDA-MB-231 and MCF-7 respectively. **(D–F)**. CCK-8 cell viability assay after DSN1 overexpression on breast cancer cell lines MDA-MB-231 and MCF-7 respectively. **(G, H)** Effect of DSN1 downregulation on colony formation. **(I, J)** Effect of DSN1 overexpression on colony formation. Each experiment was conducted three times and the results are presented as the average with the standard deviation (SD). Significance levels are denoted as follows: *p < 0.05, **p < 0.01, ***p < 0.001, ****p < 0.0001.

CCK-8 assays were used to examine the influence of DSN1 on the growth of breast cancer cells. The results showed that lower levels of DSN1 expression led to decreased cell survival in the breast cancer cell lines MDA-MB-231 and MCF-7 (**Figures 5A–C**), while overexpression of DSN1 resulted in increased cell viability (**Figures 5D–F**). Furthermore, results from colony formation assays were consistent with those from the CCK-8 assays, indicating that the proliferation of cell clones could be suppressed by transfecting siRNAs targeting DSN1 (**Figures 5G, H**). Conversely, introducing the DSN1 plasmid through transfection led to an increase in the number of cell clones (**Figures 5I, J**).

### 3.6 Effects of DSN1 on breast cancer cell proliferation, cell cycle, and apoptosis

**Figure 6:**
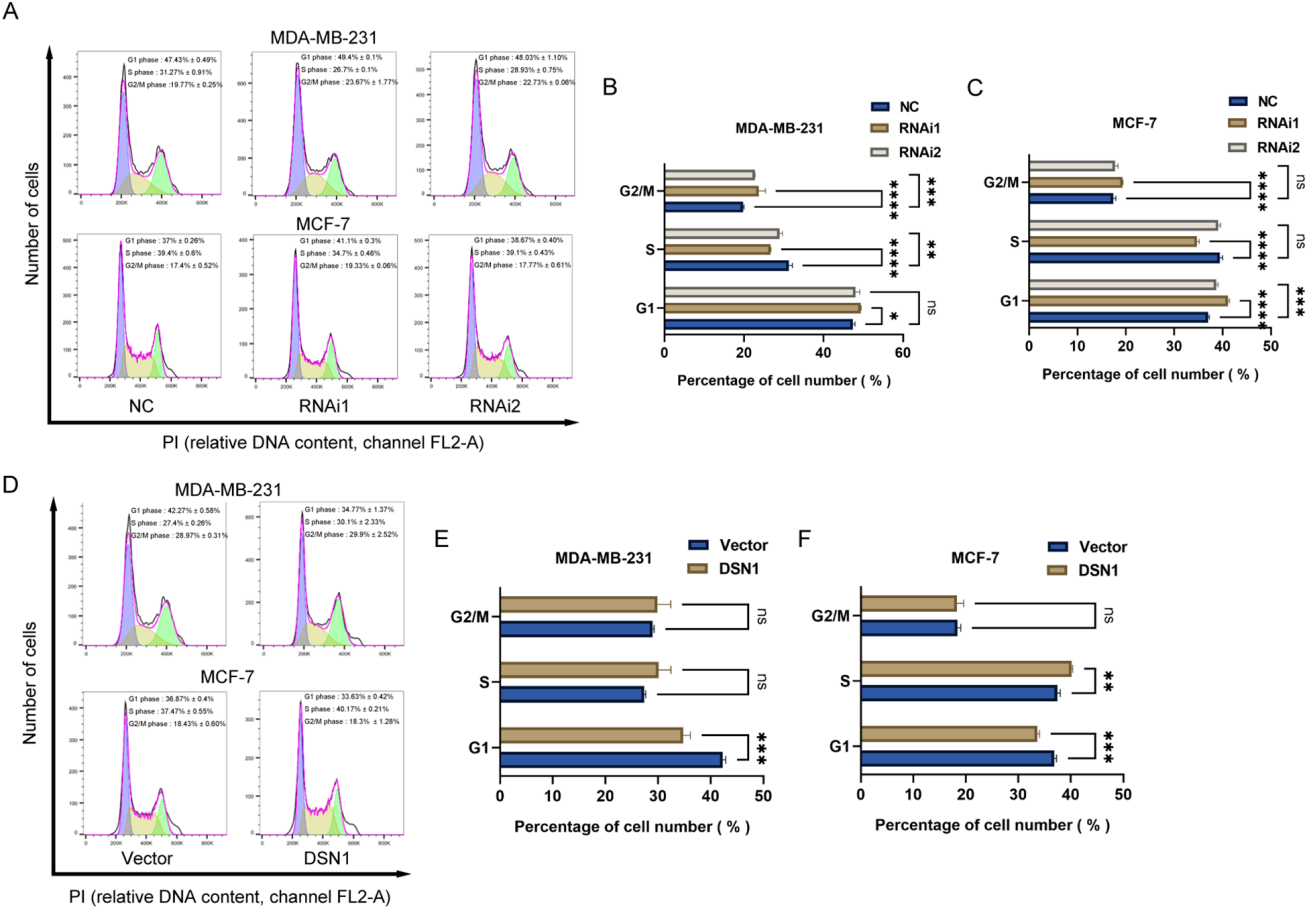
DSN1 regulates cell cycles of breast cancer cells. **(A–C)** Effect of DSN1 downregulation on cell cycles. **(D–F)** Effect of DSN1 overexpression on cell cycles. Significance levels are denoted as follows: *p < 0.05, **p < 0.01, ***p < 0.001, ****p < 0.0001.

**Figure 7:**
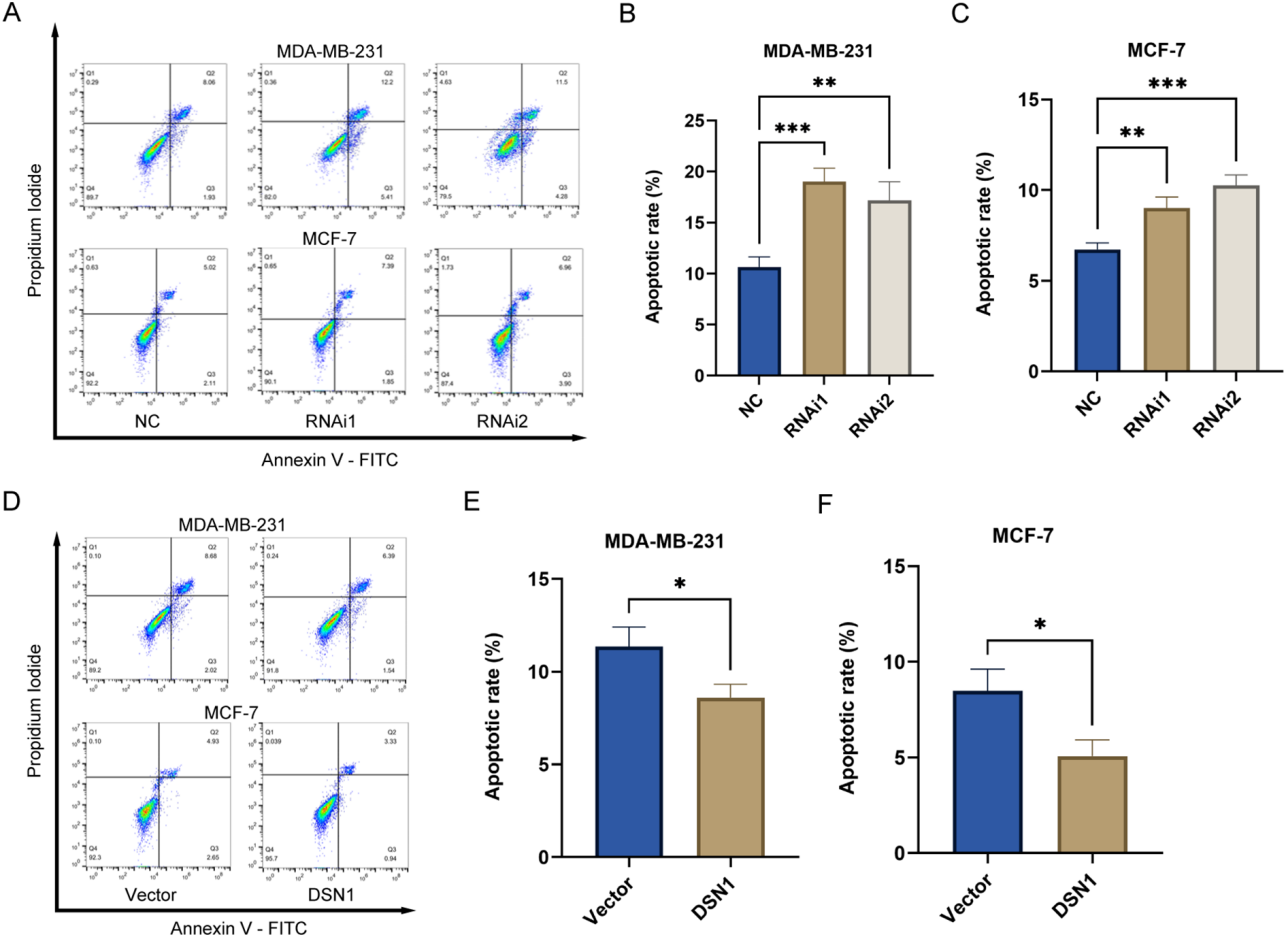
DSN1 regulates cell apoptosis of breast cancer cells. **(A-C)** DSN1 knockdown can enhance cellular apoptosis in breast cancer cells. **(D-F)** Overexpression of DSN1 inhibits cell apoptosis in breast cancer cells. Significance levels are denoted as follows: *p < 0.05, **p < 0.01, ***p < 0.001.

Flow cytometry analysis showed that there was a greater percentage of cells in the G0/G1 phase in MDA-MB-231 and MCF-7 cells transfected with DSN1 RNAi compared to the negative control group (**Figures 6A–C**). In contrast, cells transfected with the DSN1 plasmid showed a reduced percentage of cells in the G0/G1 phase (**Figures 6D–F**). Reducing DSN1 levels increased cell death in breast cancer cell lines (**Figures 7A–C**), while increasing DSN1 expression prevented cell death in breast cancer cells (**Figures 7D–F**). DSN1 silencing primarily arrested cells in G0/G1, reducing S-phase entry. Overexpression promoted G1/S transition but did not significantly alter G2/M progression, suggesting DSN1’s dominant role in early cell cycle regulation.

### 3.7 DSN1 promotes breast cancer cell proliferation through the cell cycle pathways

**Figure 8:**
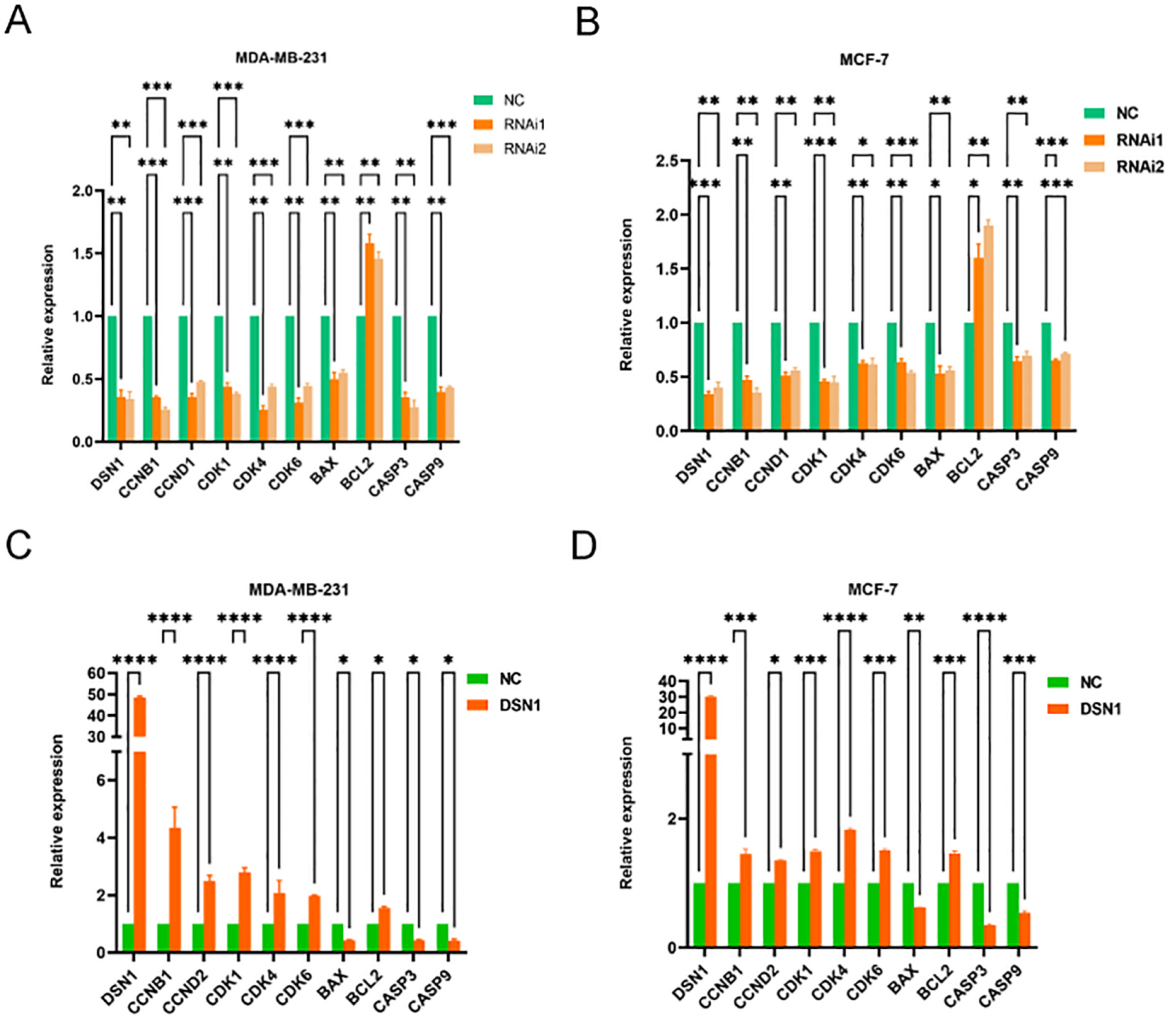
Cell cycle pathways is regulated by DSN1. **(A, B)** The impact of DSN1 knockdown on cell cycle and apoptosis-related genes on breast cancer cell lines MDA-MB-231 and MCF-7 respectively. **(C, D)** The impact of DSN1 overexpression on cell cycle and apoptosis-related genes on breast cancer cell lines MDA-MB-231 and MCF-7 respectively. Significance levels are denoted as follows: *p < 0.05, **p < 0.01, ***p < 0.001, ****p < 0.0001.

To elucidate the underlying mechanisms, the levels of cell cycle regulatory genes and apoptosis-associated genes were evaluated. **Figures 8A, B** show that reducing DSN1 in MDA-MB-231 and MCF-7 cells decreased the expression of CCNB1, CCND1, CDK1, CDK4, CDK6, BAX, CASP3, and CASP9. Conversely, overexpression of DSN1 resulted in the opposite modulation of these cell cycle and apoptosis-related proteins (**Figures 8C, D**). These findings suggest that DSN1 influences cell cycle progression by regulating key cell cycle pathways.

### 3.8 Potential therapeutic drugs for DSN1 and the molecular docking landscape on drugs against DSN1

**Figure 9:**
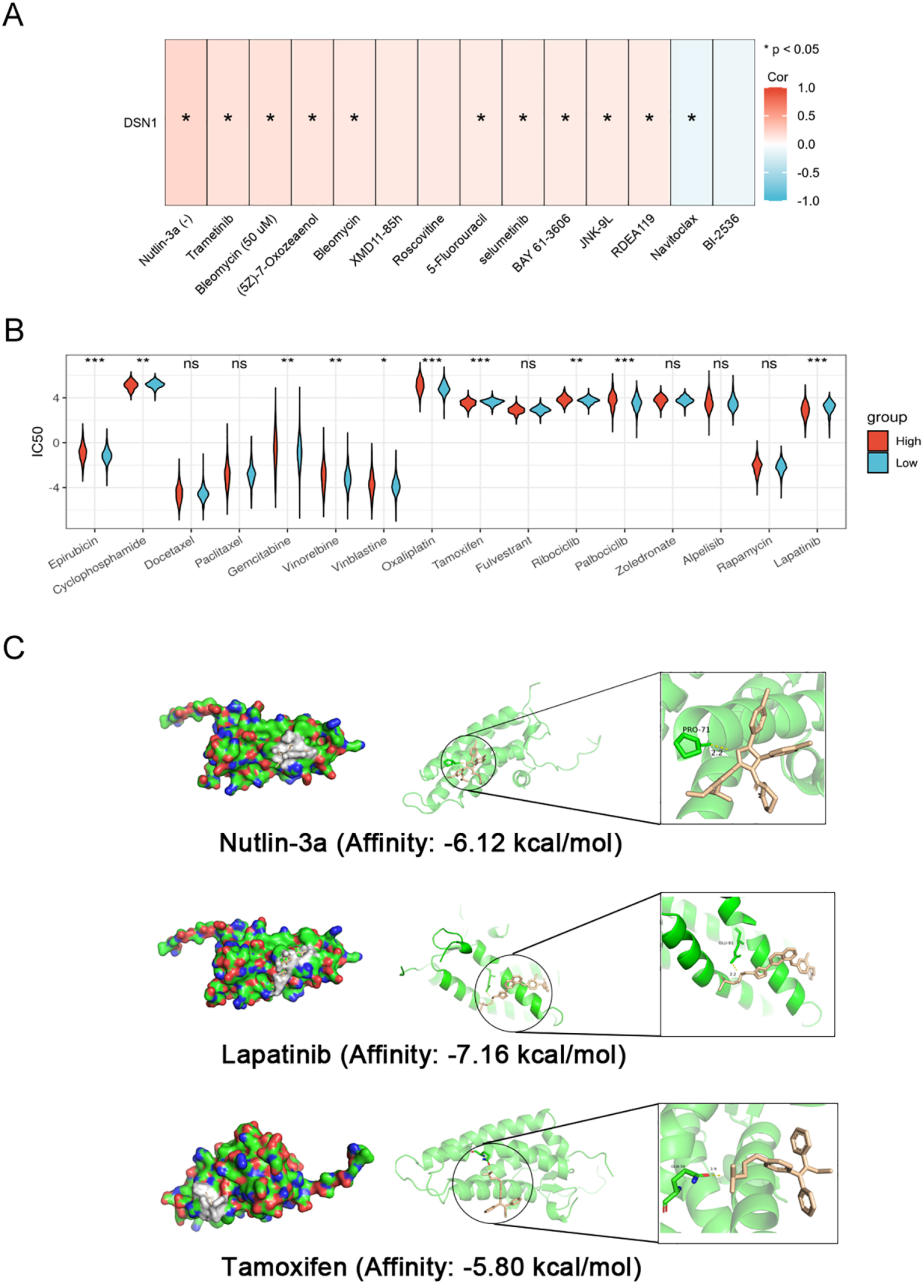
Drug sensitivity analysis based on the expression of DSN1 and molecular docking pattern of representative drugs with DSN1. **(A)** The correlation between DSN1 expression and GDSC drug sensitivity in pan-cancer. **(B)** The IC50 values of the sixteen antitumor drugs with high and low DSN1 expression groups in breast cancer. **(C)** Molecular docking pattern of DSN1with Nutlin-3a, Lapatinib and Tamoxifen respectively. Significance levels are denoted as follows: *p < 0.05, **p < 0.01, ***p < 0.001.

The drug sensitivity analysis on the GSCA website showed that in pan-cancer, DSN1 expression was positively correlated with drugs such as Nutlin-3a and 5-Fluorouracil (**Figure 9A**). Subsequently, we used the oncoPredict package to analyze the differences in drug sensitivity between high and low DSN1 expression groups in breast cancer. Drug sensitivity analysis revealed divergent responses in DSN1-high breast cancers: conventional chemotherapies (Epirubicin, Cyclophosphamide) and CDK4/6 inhibitors (Ribociclib, Palbociclib) exhibited significantly higher IC50 values in the high-DSN1 group (**Figure 9B**, p < 0.05), suggesting intrinsic resistance. Conversely, targeted agents Tamoxifen and Lapatinib showed markedly lower IC50 values in DSN1-overexpressing tumors (**Figure 9B**, p < 0.01), indicating heightened therapeutic efficacy. This dichotomy may reflect DSN1’s dual roles in cell cycle dysregulation and survival pathway modulation.”. We then did molecular docking of potential therapeutic drugs Nutlin-3a, Tamoxifen and Lapatinib with DSN1 protein, the results are shown in **Figure 9C**, which each drug candidates bound to its protein targets through visible hydrogen bonds and strong electrostatic interactions. Moreover, the hydrophobic pockets of each target were occupied successfully by the three drugs. Additionally, they all have low binding energy of -6.12, -7.16 and -5.8 kcal/mol, indicating highly stable binding. These results suggest that Nutlin-3a, Lapatinib and Tamoxifen are potentially effective agents for DSN1-overexpressing breast cancers.

## 4 Discussion

**Figure 10:**
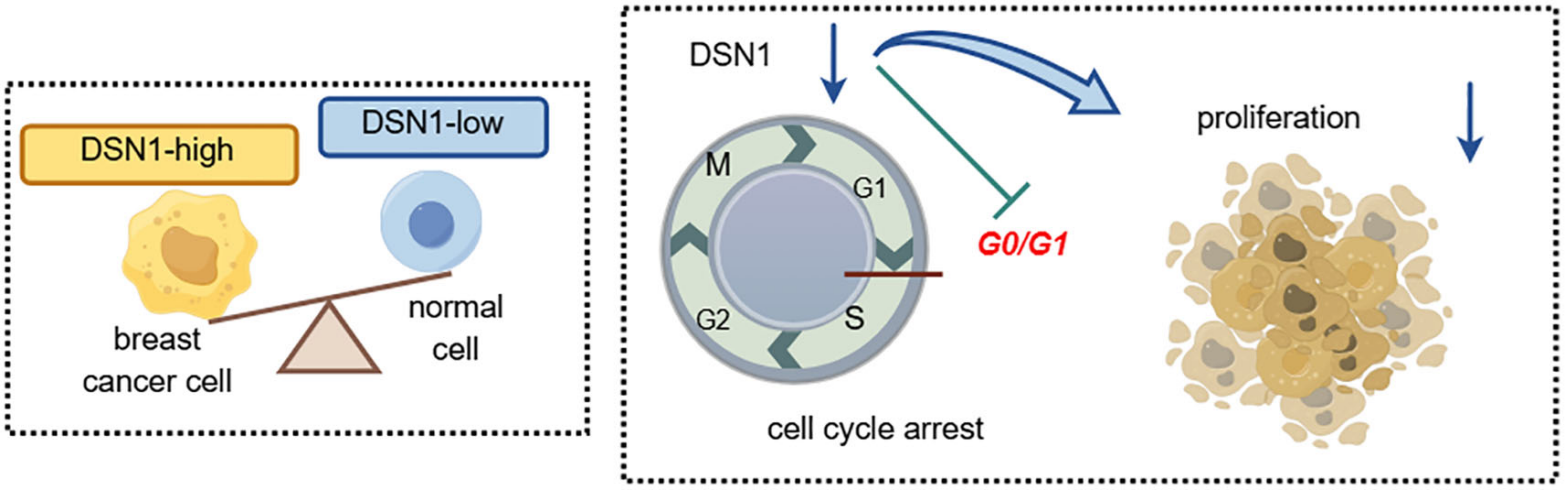
Graphic abstract. DSN1 is abundantly present in breast cancer, and this elevated presence frequently linked to the activation of genes involved in cell growth, facilitating the proliferation of breast cancer cells by regulating cell cycle pathways.

Breast cancer is a highly heterogeneous tumor, and despite advancements in treatment options, it remains a significant public health concern (23, 24). Discovering molecules that are essential for the onset and progression of breast cancer, along with identifying molecular indicators linked to its diagnosis and therapy, is urgent needed (25). Our study found that DSN1 is abundantly present in breast cancer, and this elevated presence frequently correlates with unfavorable clinical outcomes. Moreover, increased levels of DSN1 are closely linked to the activation of genes involved in cell growth, facilitating the proliferation of breast cancer cells by regulating cell cycle pathways (**Figure 10**). This suggests that DSN1 could be a potential target for diagnosing and treating breast cancer.

DSN1 is essential for kinetochore organization and stability, ensuring accurate chromosome replication. Dysfunction in kinetochore proteins can cause chromosomal misalignment, abnormal polyploidy, and mutations, leading to genomic instability (7, 26, 27). Chromosomal instability is a significant contributor to tumors, and abnormal kinetochore function is one of the primary causes of chromosomal instability (28). Tumors with combined chromosomal instability also contribute to tumor heterogeneity and treatment failure (29, 30). Previous research has demonstrated high DSN1 expression in colorectal cancer, osteosarcoma, and other cancers (11–13, 31–34). Our study has also identified elevated DSN1 expression in breast cancer, with varying expression patterns among patients with different molecular subtypes, particularly more pronounced in luminal type patients and those with advanced tumor stages. This indicates that DSN1 could be more significant in estrogen receptor-positive breast cancer and have a more crucial impact on individuals with advanced breast cancer. Currently, the diagnosis of breast cancer relies mainly on imaging and pathological methods, with no suitable molecular markers available (35, 36). DSN1 can serve as a discriminative marker between breast cancer tissues and normal tissues, exhibiting an AUC of 0.885, potentially surpassing existing tumor markers like CA153 and HER2, making it a promising molecular marker for breast cancer diagnosis.

Our immunohistochemical analysis indicates a higher proportion of DSN1 expression in the high Ki67 expression group, aligning with our findings from the TCGA database, which show a strong correlation between DSN1 expression and cell proliferation-related genes such as AURKA and PCNA. Studies have revealed abnormal expression of Ki67, PCNA, and AURKA in breast cancer, all closely associated with disease progression, implying a close relationship between DSN1 and cell proliferation in breast cancer (37–41). Therefore, we conducted further experiments to verify that breast cancer cells with high DSN1 expression exhibit enhanced proliferation capabilities, while interfering with DSN1 expression effectively reduces cell proliferation. Notably, inhibition of DSN1 via siRNA knockdown significantly induced cellular apoptosis, suggesting that targeting DSN1 promotes programmed cell death in breast cancer cells. GSEA analysis demonstrates that genes in the high DSN1 expression group are enriched in DNA replication and cell cycle-related functions and pathways. Consequently, we conducted experiments to confirm the influence of DSN1 on the cell cycle, demonstrating that suppressing DSN1 can halt the cell cycle in the G0/G1 stage, leading to decreased cell growth. Conversely, overexpression of DSN1 allows breast cancer cells to bypass G0/G1 phase arrest, resulting in a higher number of cells in the S and G2/M phases, ultimately enhancing cell proliferation. The transcriptional regulatory network of DSN1 was initially explored using a candidate gene approach based on pathway enrichment. To obtain a comprehensive and unbiased view, future studies will employ RNA-sequencing to define the global transcriptomic changes induced by DSN1 modulation.

DNA replication serves as the foundation for orderly mitosis in cells, and the accurate separation of chromosomes is crucial for ensuring precise DNA replication (42). Cell division involves the orderly duplication and splitting of cells, requiring the regulation of multiple proteins such as cell cycle regulators, CDKs, oncogenes, and tumor suppressor genes (43, 44). The progression of the cell cycle is a result of continuous interaction between cell cycle proteins and CDKs (45, 46). Cyclin D1 plays a crucial part in the transition from G1 to the S phase. Cyclin D1 binds to CDK4/6, phosphorylates Rb, and dissociates from the E2F promoter region, leading to the activation of genes associated with cell growth and development (47, 48). Consequently, we examined core proteins related to the cell cycle such as CCND1, CCNB1, CDK1, CDK4, and CDK6 and observed that their expression is heightened when DSN1 is overexpressed but diminishes when DSN1 expression is inhibited. It should be noted that the impact of DSN1 on the protein levels of key cell cycle regulators was not determined in this study. Future work will include Western blot analysis to confirm whether the observed transcriptional changes translate to corresponding alterations in protein expression. The results suggest that DSN1 enhances the advancement of breast tumors by affecting cell cycle pathways, making it a promising target for treating breast cancer. Our data robustly demonstrate that DSN1 is a critical promoter of breast cancer cell proliferation, primarily through its direct regulation of key cell cycle molecules (e.g., Cyclins, CDKs), whereas the increase in apoptosis, although statistically significant, appears to be a downstream outcome of disrupted cycle progression, not necessarily the direct target of DSN1. The concurrent dysregulation of key apoptotic genes (Bax, Casp3, Bcl2) upon DSN1 modulation suggests a complex interplay. While the significant increase in apoptosis upon DSN1 knockdown is clear, its exact mechanism warrants further investigation. It is plausible that the profound cell cycle arrest induced by DSN1 depletion leads to cellular stress or DNA damage, thereby triggering apoptosis as a downstream event, rather than through direct transcriptional regulation of apoptotic pathways by DSN1. Considering the usage of CDK4/6 inhibitors in breast cancer treatment and the absence of drug markers, we intend to investigate the correlation between DSN1 expression and CDK4/6 inhibitors as well as potential combination therapy strategies following the development of resistance to CDK4/6 inhibitors (49–51). It is important to note that while we observed concomitant G0/G1 phase arrest and downregulation of key cyclins and CDKs upon DSN1 knockdown, our current data cannot unequivocally determine if this is a direct transcriptional regulation by DSN1 or an indirect consequence of the cell cycle blockade. Further mechanistic studies, such as chromatin immunoprecipitation (ChIP) assays, are warranted to elucidate the direct transcriptional targets of DSN1. Additionally, our research has identified an abundance of DSN1 expression in E2F targets and the PI3K/AKT/MTOR signaling pathway. E2Fs play a crucial role in controlling the production of cell cycle-related proteins and DNA replication, which can result in instability of chromosomes (52, 53). E2F1 in breast cancer can promote the advancement of breast cancer through multiple pathways (54). The PI3K/AKT/MTOR pathway plays a crucial role in cell growth, and targeting PI3K and MTOR is a key strategy for treating advanced breast cancer (55). Hence, we plan to delve deeper into the direct role of DSN1 in breast cancer through E2F1 and the PI3K/AKT/MTOR pathway, as well as examine possible combined treatment approaches.

Our subsequent drug sensitivity analysis indicated that high DSN1 expression may be one of the causes of breast cancer resistance to some chemotherapeutic drugs such as Epirubicin, Cyclophosphamide, However, the sensitivity to Tamoxifen and Lapatinib would be a bit higher. Nutlin-3a is an inhibitor of MDM2, which plays an important role in anti-tumor cell proliferation (56). Our drug sensitivity and molecular docking analyses also indicated that Nutlin-3a has a role with DSN1 and may be a potential therapeutic agent. In addition, we noticed that the IC50 of CDK4/6 inhibitors was higher in the DSN1 high-expression group than in the low-expression group, which suggests that the signaling pathways involved in DSN1 are more complex and deserve further exploration.

While our study provides compelling multi-omics and *in vitro* evidence for the oncogenic role of DSN1 in breast cancer, it is not without limitations. First, our conclusions regarding the association between DSN1 expression and drug sensitivity (e.g., to Tamoxifen and Lapatinib) are primarily derived from bioinformatic analyses and molecular docking predictions. Although these computational approaches are widely used and provide strong hypothesis-generating evidence, they lack experimental validation. We acknowledge that direct *in vitro* determination of IC50 values in DSN1-modulated cells is necessary to conclusively establish DSN1 as a mediator of chemoresistance. Therefore, performing these drug sensitivity assays constitutes an immediate and critical next step in our future research plans. Similarly, future work will employ co-immunoprecipitation (Co-IP) assays to validate the predicted physical interaction between DSN1 and CDK4/CCND1, and rescue experiments will be critical to formally establish the causal relationship within this pathway. Despite these limitations, our current findings lay a solid foundation for these subsequent investigations and highlight DSN1 as a promising novel therapeutic target worthy of further exploration.

## 5 Conclusion

To sum up, this research clarifies the elevated levels of DSN1 in breast cancer and its correlation with negative clinical pathological characteristics, suggesting that DSN1 could enhance the growth of breast cancer cells through the control of cell cycle and apoptosis. Our data, combined with bioinformatic predictions, suggest that DSN1 may promote CDK4/6 inhibitor resistance by upregulating CCND1, but this hypothesis requires direct validation through combination treatment experiments. We further find that Nutlin-3a, Lapatinib and Tamoxifen are potentially effective agents for DSN1-overexpressing breast cancers. Bioinformatic analyses suggest a potential correlation between high DSN1 expression and reduced drug sensitivity, which warrants further experimental investigation. By overcoming these constraints, we can gain a deeper insight into the function of DSN1 in breast cancer and create improved diagnostic and treatment approaches.

## Data Availability

The original contributions presented in the study are included in the article/**Supplementary Material**. Further inquiries can be directed to the corresponding authors.

## Supplementary material

Supplementary Figure 1 Validation of DSN1 knockdown and overexpression efficiency in MDA-MB-231 and MCF-7 cell lines. MDA-MB-231 and MCF-7 cells were transfected with (siRNA) or (plasmid DNA) to achieve knockdown or overexpression of DSN1, respectively. **(A, B)** Immunoblots showing DSN1 protein levels in both cell lines. GAPDH was used as an internal control.

Supplementary Figure 2 Prognostic value of DSN1 expression in breast cancer and its molecular subtypes. **(A)** Kaplan-Meier curves for overall survival (OS) of all patients from the TCGA-BRCA cohort stratified by high and low DSN1 expression **(B–D)** Subgroup analysis of OS based on molecular subtypes: **(B)** Luminal A and Luminal B, **(C)** HER2-positive, and **(D)** Basal-like subtypes.
