# Supplementary figures and images for: DSN1 drives breast cancer progression via cell cycle regulation: diagnostic and therapeutic implications
Source: Front Oncol. 2026 Jan 20;15:1711214. doi: 10.3389/fonc.2025.1711214 (PMC12864087; doi:10.3389/fonc.2025.1711214)

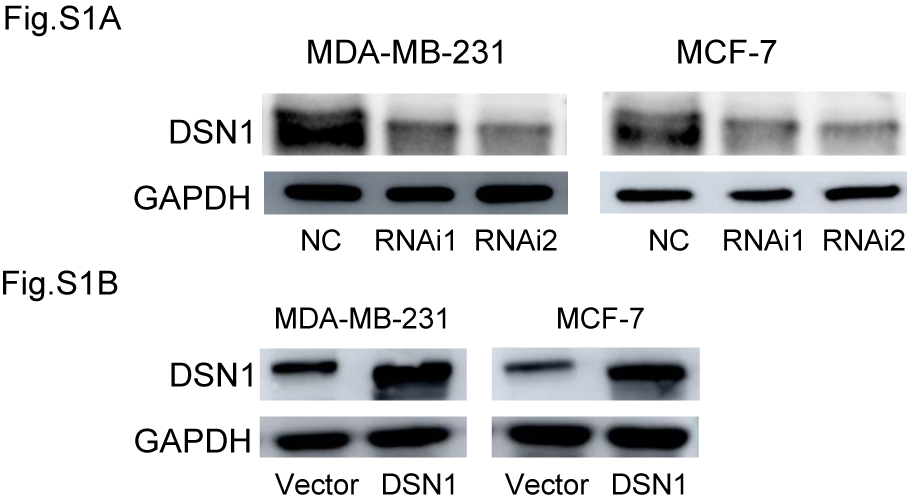

Supplement: Supplementary Figure 1 — Validation of DSN1 knockdown and overexpression efficiency in MDA-MB-231 and MCF-7 cell lines. MDA-MB-231 and MCF-7 cells were transfected with (siRNA) or (plasmid DNA) to achieve knockdown or overexpression of DSN1, respectively. (A, B) Immunoblots showing DSN1 protein levels in both cell lines. GAPDH was used as an internal control. [file Image1.tif]

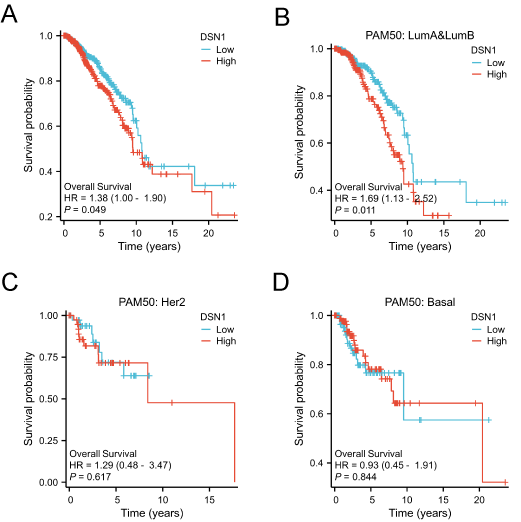

Supplement: Supplementary Figure 2 — Prognostic value of DSN1 expression in breast cancer and its molecular subtypes. (A) Kaplan-Meier curves for overall survival (OS) of all patients from the TCGA-BRCA cohort stratified by high and low DSN1 expression (B–D) Subgroup analysis of OS based on molecular subtypes: (B) Luminal A and Luminal B, (C) HER2-positive, and (D) Basal-like subtypes. [file Image2.tif]
